# Phenotypic and molecular characterization of *Staphylococcus aureus *isolates expressing low- and high-level mupirocin resistance in Nigeria and South Africa

**DOI:** 10.1186/1471-2334-9-10

**Published:** 2009-01-28

**Authors:** Adebayo O Shittu, Edet E Udo, Johnson Lin

**Affiliations:** 1 Department of Microbiology, Obafemi Awolowo University, Ile-Ife, Nigeria; 2 Department of Microbiology, Faculty of Medicine, Kuwait University, Kuwait City, Kuwait; 3 School of Biochemistry, Genetics and Microbiology, University of KwaZulu-Natal (Westville Campus), Private Bag X54001, Durban, Republic of South Africa

## Abstract

**Background:**

Mupirocin is a topical antimicrobial agent which is used for the treatment of skin and postoperative wound infections, and the prevention of nasal carriage of methicillin-resistant *Staphylococcus aureus *(MRSA). However, the prevalence of mupirocin resistance in *S. aureus*, particularly in MRSA, has increased with the extensive and widespread use of this agent in hospital settings. This study characterized low- and high-level mupirocin-resistant *S. aureus *isolates obtained from Nigeria and South Africa.

**Methods:**

A total of 17 mupirocin-resistant *S. aureus *isolates obtained from two previous studies in Nigeria and South Africa, were characterized by antibiogram, PCR-RFLP of the coagulase gene and PFGE. High-level mupirocin resistant isolates were confirmed by PCR detection of the *mupA *gene. The genetic location of the resistance determinants was established by curing and transfer experiments.

**Results:**

All the low-level mupirocin resistant isolates were MRSA and resistant to gentamicin, tetracycline and trimethoprim. PFGE identified a major clone in two health care institutions located in Durban and a health care facility in Pietermaritzburg, Greytown and Empangeni. Curing and transfer experiments indicated that high-level mupirocin resistance was located on a 41.1 kb plasmid in the South African strain (A15). Furthermore, the transfer of high-level mupirocin resistance was demonstrated by the conjugative transfer of the 41.1 kb plasmid alone or with the co-transfer of a plasmid encoding resistance to cadmium. The size of the mupirocin-resistance encoding plasmid in the Nigerian strain (35 IBA) was approximately 35 kb.

**Conclusion:**

The emergence of mupirocin-resistant *S. aureus *isolates in Nigeria and South Africa should be of great concern to medical personnel in these countries. It is recommended that methicillin-susceptible *S. aureus *(MSSA) and MRSA should be routinely tested for mupirocin resistance even in facilities where the agent is not administered. Urgent measures, including judicious use of mupirocin, need to be taken to prevent clonal dissemination of the mupirocin/methicillin resistant *S. aureus *in KZN, South Africa and the transfer of the conjugative plasmid encoding high-level mupirocin resistance identified in this study.

## Background

The treatment of infections caused by antibiotic-resistant bacteria especially methicillin-resistant *Staphylococcus aureus *(MRSA) has become a worldwide problem in hospital and community settings. Infection control programmes implement measures to contain the dissemination of MRSA which include efforts to eradicate carriage of *S. aureus *[1]. The antibiotic mupirocin used topically has been shown to possess potent activity against staphylococci and is used for the treatment of skin and postoperative wound infections, and the prevention of nasal carriage of MRSA [2,3]. However the widespread use of mupirocin led to resistance in *S. aureus*, which has been reported worldwide [4-9].

Mupirocin-resistant strains are divided into two groups: low- and high-level resistance (MIC 8–256 and >256 mg/L, respectively) [2]. In most cases, low-level resistance to mupirocin is related to alterations in the host isoleucyl-tRNA synthetase (IRS) [10,11]. Until recently, chromosomal mupirocin resistance was considered clinically unimportant [2,12]. However, low-level mupirocin resistance appears to be more prevalent in clinical isolates than high-level resistance [13-15], and the emergence of low-level mupirocin resistance has been shown to increase failure rates for nasal decolonization of MRSA [16-18]. High-level mupirocin resistant strains cannot be eradicated with mupirocin and constitute a serious clinical problem, especially when they are resistant to methicillin [19]. The clinical isolates exhibiting high-level resistance to mupirocin contain two distinct IRS enzymes: endogenous IRS plus an additional IRS encoded by the *mupA *gene [20], which is carried on plasmids that vary in size, restriction patterns and their ability to be transferred in conjugation experiments [4,20-23]. The *mupA *gene has also been reported in the genomic DNA of a few *S. aureus *isolates expressing low-level resistance, suggesting that the *mupA *gene may be located in the chromosome [24,25]. Moreover, the chromosomal location of the *mupA *gene in *S. aureus *expressing high-level mupirocin resistance has been described [26].

The previous studies conducted by these investigators indicated that the prevalence of *S. aureus *resistance to mupirocin in South Africa and Nigeria was 7% and 0.5% respectively [7,27]. This study reports on the phenotypic and molecular characterization of mupirocin-resistant *S. aureus *isolates in Nigeria and South Africa.

## Methods

### Antimicrobial susceptibility testing and PCR detection of the mupA gene

A total of 17 *S. aureus *isolates were investigated based on their resistance to mupirocin from two studies on antibiotic susceptibility patterns of *S. aureus *obtained from clinical samples in Nigeria and South Africa [7,27]. Susceptibility to various antibiotics was based on the disk diffusion method according to the National Committee for Clinical Laboratory Standards (now Clinical Laboratory Standards Institute) guidelines [28]. Susceptibility to heavy metals (cadmium acetate, mercuric chloride) and nucleic-acid binding compounds (ethidium bromide and propamidine isethionate) was performed on the isolates using disks prepared in the laboratory with the indicated concentrations (10 μl): cadmium acetate (50 μg), propamidine isethionate (50 μg), mercuric chloride, (109 μg) and ethidium bromide (60 μg). Interpretation of zone diameters were considered as follows: ≤ 9 mm (resistance), 10–12 mm (intermediate) for cadmium acetate; ≤ 25 mm (resistance) for mercuric chloride; ≤ 10 mm (resistance), 11–14 mm (intermediate) for propamidine isethionate; and ≤ 9 mm (resistance), 10–14 mm (intermediate) for ethidium bromide, as published previously [29]. Minimum inhibitory concentration (MIC) of mupirocin was determined using E-test strips (AB Biodisk, Solna, Sweden) according to the manufacturer's instructions. The high-level mupirocin-resistant isolates (based on the disk diffusion and E-test methods) were confirmed by PCR detection of the *mupA *gene as described previously [7,27].

The low-level mupirocin-resistant isolates were obtained from eleven wound samples and one isolate each from blood, urine samples, and endotracheal aspirate. Furthermore, the high-level mupirocin-resistant isolate (A15) from South Africa was obtained from a wound sample while 35 IBA, the isolate from Nigeria was recovered from a blood sample. Information on the source of the methicillin-susceptible *S. aureus *(MSSA) from South Africa (P1929) was not available.

### Plasmid DNA isolation

Plasmid DNA was isolated by the cetyl trimethyl ammonium bromide method (CTAB) as earlier reported [30]. Plasmids were analysed by agarose (0.6% w/v) gel electrophoresis in 1 × TAE buffer (pH 7.2) at 25 V for 16 hr. The *Staphylococcus aureus *strain WBG 4483, which has 4 plasmids (40.3 kb, 22.5 kb, 4.4 kb and 3.5 kb) served as the plasmid molecular size standard. The approximate plasmid sizes (closed circular forms) were estimated by visual inspection and using the GeneTools program (SynGene Bioimaging System, Cambridge, United Kingdom).

### Curing experiments

Two of the three high-level mupirocin-resistant isolates (A15 – South Africa and 35 IBA – Nigeria) were selected for curing and conjugation experiments based on their antibiotic resistance profile. The mupirocin-resistant isolate P1929 from South Africa was not included in the experiment because its antibiotic susceptibility pattern was similar to 35 IBA. The loss of resistance determinants (plasmids) was investigated as previously reported [31]. The isolates were subcultured on Brain Heart Infusion Agar (BHIA, Biolab, South Africa) and incubated at 43.5°C for 24 hr. Sub-culturing on freshly prepared BHIA was performed twice and incubated as stated above. Thereafter, serial dilutions of the culture were plated on BHIA plates, and incubated at 37°C for 24 and 48 hrs. Single colonies were replica plated on BHIA plates as control and selection plates of BHIA containing mupirocin (10 mg/L) and erythromycin (5 mg/L) for A15, and mupirocin (10 mg/L) for 35 IBA. Single colonies which grew on the control plate but did not grow on the selection plates were noted and antibiotic susceptibility testing was performed on them to verify loss of resistance. Thereafter, colonies were screened for loss of the resistance determinants by plasmid analysis and visualized after electrophoresis on 0.6% agarose gels. Cured strains (susceptible to mupirocin) were confirmed for loss of resistance by a negative result for the *mupA *gene by PCR.

### Conjugation Experiments

Conjugation experiments were performed in Brain Heart Infusion Broth (BHIB, Biolab, South Africa) with 40% polyethylene glycol as previously reported [31]. Strains A15 and 35 IBA were used as donors and *S. aureus *WBG541 (fusidic and rifampicin-resistant) was the recipient strain. Donor and recipients controls were set up with each test. Transfer was considered to have occurred when growth was observed on the selection plates from the donor-recipient mixtures and not from the control experiments. The transconjugants (using A15 as the donor strain) were screened on BHIA plates containing mupirocin (10 mg/L), and fusidic acid (5 mg/L); erythromycin (5 mg/L) and fusidic acid (5 mg/L). Selections of transconjugants for 35 IBA were made on BHIA containing mupirocin (10 mg/L) and fusidic acid (5 mg/L); tetracycline (5 mg/L) and fusidic acid (5 mg/L). Transfer frequency was expressed as the number of transconjugants per number of donor cells. Single colonies of transconjugants were screened on BHIA containing appropriate antibiotics by the replica plating method and antibiotic susceptibility testing was performed on transconjugants. Furthermore, the transconjugants were screened to confirm plasmid content by agarose gel electrophoresis. In addition, transconjugants exhibiting high-level resistance to mupirocin were confirmed by the MIC values and detection of the *mupA *gene.

### PCR-RFLP of the coagulase gene

The 3' end region of the coagulase gene was amplified by PCR and restriction fragment polymorphisms (RFLPs) of the amplicons were determined by digestion with *Alu*I (Fermentas, UK) as previously described [7].

### PFGE typing

PFGE typing of *Sma*I (Fermentas, UK) digested DNA was carried out by a modification of the protocol previously described [32]. The banding patterns were interpreted visually and strains showing the same PFGE pattern were classified into pulsotypes using an alphabet (e.g. A, B, C etc). Numeric sub-codes were used to represent < 3 band difference (subtypes, e.g. A1, B1, etc) based on a previous report [33].

## Results

### Resistance pattern of low and high-level mupirocin resistant S. aureus isolates to antimicrobial agents

The resistance profiles of the mupirocin-resistant *S. aureus *isolates to various antimicrobial agents are presented in Table 1. A total of 14 isolates from six health care institutions in KZN, South Africa exhibited low-level resistance to mupirocin (MIC: 8–24 mg/L). They were also resistant to methicillin, gentamicin, tetracycline and trimethoprim. A total of 12 low-level mupirocin resistant isolates were resistant to erythromycin, 10 isolates were resistant to ciprofloxacin, and four isolates each were resistant to rifampicin and chloramphenicol. Resistotyping revealed that six low-level mupirocin resistant isolates expressed resistance to cadmium acetate, propamidine isethionate, mercuric chloride and ethidium bromide. Four isolates were resistant only to mercuric chloride and two isolates were resistant to cadmium acetate and mercuric chloride. An MRSA isolate was susceptible to the heavy metals and nucleic-acid binding compounds. The *mupA *gene was not detected in the low-level mupirocin resistant strains while the high-level mupirocin resistant isolates with MIC of >1024 mg/L tested positive for the *mupA *gene. Furthermore, the high-level mupirocin-resistant isolates were resistant to cadmium acetate but susceptible to mercuric chloride and nucleic acid binding compounds (Table 1).

**Table 1:** Characterization (antibiogram, PCR-RFLP of the coagulase gene and PFGE) of low and high-level mupirocin resistant *S. aureus *isolated in Nigeria and South Africa

Strain No	Antibacterial resistance patterns	MUP MIC (mg/L)	*mupA * gene	Coagulase gene (± 20 bp)	PCR-RFLP Coagulase gene (bp)	PFGE type
A1	PEN, OX, GN, ERY, CHL, TET, TS, CIP, MU (L), Hg	12	-	800	324, 405	A

A2	PEN, OX, GN, TET, TS, RF, MU (L)	24	-	650	81, 567	B

A3	PEN, OX, GN, ERY, TET, TS, CIP, MU (L), Cad, Pi, Hg, Eb	16	-	850	324, 405	A

A4	PEN, OX, GN, TET, TS, RF, MU (L), Cad, Hg	24	-	650	81, 567	B

A5	PEN, OX, GN, ERY, CHL, TET, TS, CIP, MU (L), Cad, Pi, Hg, Eb	12	-	800	324, 405	A1

A6	PEN, OX, GN, ERY, TET, TS, CIP, MU (L), Cad, Pi, Hg, Eb	8	-	800	81, 324, 405	A

A7	PEN, OX, GN, ERY, TET, TS, RF, MU (L), Cad	24	-	650	81, 567	B1

A8	PEN, OX, GN, ERY, CHL, TET, TS, CIP, MU (L), Cad, Pi, Hg, Eb	8	-	800	81, 324, 405	A

A9	PEN, OX, GN, ERY, TET, TS, RF, MU (L), Cad, Hg	24	-	650	81, 567	B2

A10	PEN, OX, GN, ERY, CHL, TET, TS, CIP, MU (L), Hg	12	-	800	81, 324, 405	A

A11	PEN, OX, GN, ERY, TET, TS, CIP, MU (L), Cad, Pi, Hg, Eb	8	-	800	81, 324, 405	A

A12	PEN, OX, GN, ERY, TET, TS, CIP, MU (L), Hg	12	-	850	81, 324, 405	A2

A13	PEN, OX, GN, ERY, TET, TS, CIP, MU (L), Hg	8	-	800	81, 324, 405	A1

A14	PEN, OX, GN, ERY, TET, TS, CIP, MU (L), Cad, Pi, Hg, Eb	12	-	800	81, 324, 405	A

A15	PEN, OX, GN, ERY, TET, TS, RF, MU (H), Cad	>1024	+	650	81, 567	B3

P1929	TM, TET, MU (H), Cad	>1024	+	750	243, 486	C

35 IBA	TET, MU (H), Cad	>1024	+	800	81, 162, 567	D

KEY. PEN – Penicillin, OX – Oxacillin, GN – Gentamicin, ERY – Erythromycin, CHL – Chloramphenicol,

TET – Tetracycline, TS – Trimethoprim, RF – Rifamipicin, CIP – Ciprofloxacin,

MU (H) – High-level mupirocin resistance, MU (L) – Low-level mupirocin resistance

Heavy metals – Cad: Cadmium acetate; Hg: Mercuric chloride Nucleic acid binding compounds – Eb: Ethidium bromide; Pi: Propamidine isethionate

### Genotyping (PCR-RFLP of the coagulase gene, PFGE) of low and high-level mupirocin resistant S. aureus strains

**Figure 1:**
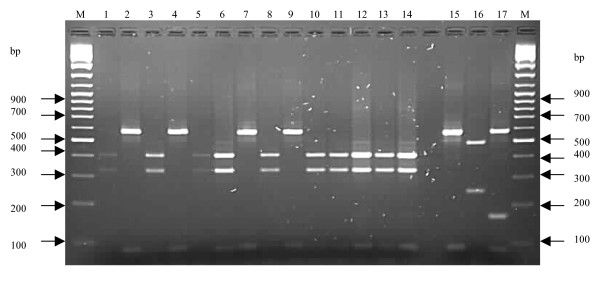
**PCR-RFLP of the coagulase gene in mupirocin-resistant strains**. M: Molecular weight marker (100 bp). Lanes 1–14: Low-level mupirocin resistant strains: A1–A14. Lanes 15–17: High-level mupirocin-resistant strains: A15, P1929 (South Africa), 35 IBA (Nigeria).

**Figure 2:**
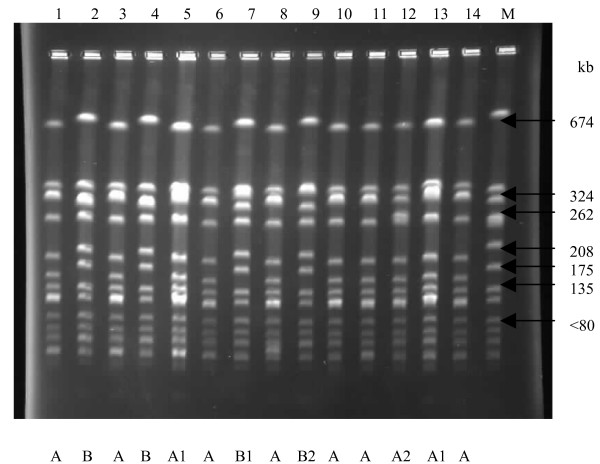
**PFGE patterns of low-level mupirocin-resistant strains**. M: Molecular weight standard *S. aureus *NCTC 8325. Lanes 1–14: A1–A14.

**Figure 3:**
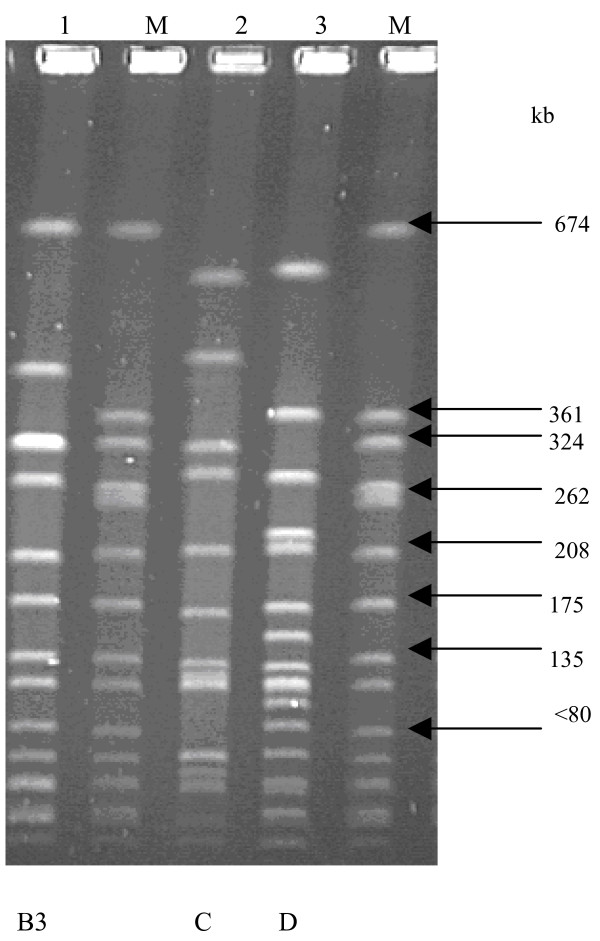
**PFGE patterns of high-level mupirocin-resistant strains from Nigeria and South Africa**. M: Molecular weight standard *S. aureus *NCTC 8325; Lane 1: A15; Lane 2: P1929; Lane 3: 35 IBA

Molecular typing by PCR-RFLP of the coagulase gene identified three RFLP patterns in the low- (81 bp, 567 bp; 325 bp, 405 bp and 81 bp, 324 bp, 405 bp) and high-level (81 bp, 567 bp; 243 bp, 486 bp and 81 bp, 162 bp, 567 bp) mupirocin-resistant strains (Figure 1). PFGE identified two pulsotypes (A and B) among the low-level mupirocin-resistant *S. aureus *strains, and 10 of 14 strains (71.4%) were classified in type A (Figure 2). The strains in type A were observed in two health care institutions located in Durban and a health care facility in Pietermaritzburg, Greytown and Empangeni, while type B was noted in two health care institutions in Durban and in Eshowe. Based on their PFGE patterns, the high-level mupirocin-resistant strains from the two countries were unrelated (Figure 3).

### Curing experiment

**Figure 4:**
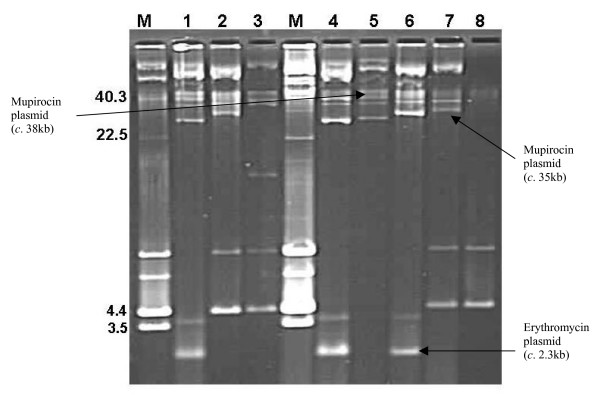
**Plasmid profiles of parent and cured strains**: M: Molecular weight marker (WBG 4483; Only closed circular DNA are labelled); Lanes 1–3: High-level mupirocin-resistant strains: Lane 1 – A15 (South Africa), Lane 2 – 35 IBA (Nigeria), Lane 3 – P1929 (South Africa); Lane 4: A15 parent strain; Lane 5 – A15^c^A (cured strain of A15 – resistant to mupirocin, susceptible to erythromycin – 38 kb plasmid encoding high-level mupirocin resistance); Lane 6 – A15^c^D (cured strain of A15 – susceptible to mupirocin, resistant to erythromycin – 2.3 kb plasmid); Lane 7 – 35 IBA parent (35 kb plasmid encoding high-level mupirocin resistance); Lane 8 – 35 IBA (CUR) – cured strain of 35 IBA (susceptible to mupirocin, resistant to tetracycline).

The isolates 35 IBA and A15 expressing high-level mupirocin resistance were subjected to curing experiments as a first step to determining the genetic location of their resistance determinants. Results of the curing experiments with 35 IBA showed that 6 of 293 colonies (2.0%) screened for loss of resistance were susceptible to mupirocin. Agarose gel electrophoresis indicated that this was associated with the loss of *c*. 35 kb plasmid (Figure 4). Of the 294 colonies screened on mupirocin and erythromycin selection plates for A15, three and six colonies lost resistance to erythromycin and mupirocin respectively. Resistance to these antibiotics in the cured strains was lost together with plasmids of *c. *2.3 kb and 38 kb respectively (Figure 4). The *mupA *gene was not detected in the cured strains (susceptible to mupirocin) by PCR.

### Conjugation experiment

**Figure 5:**
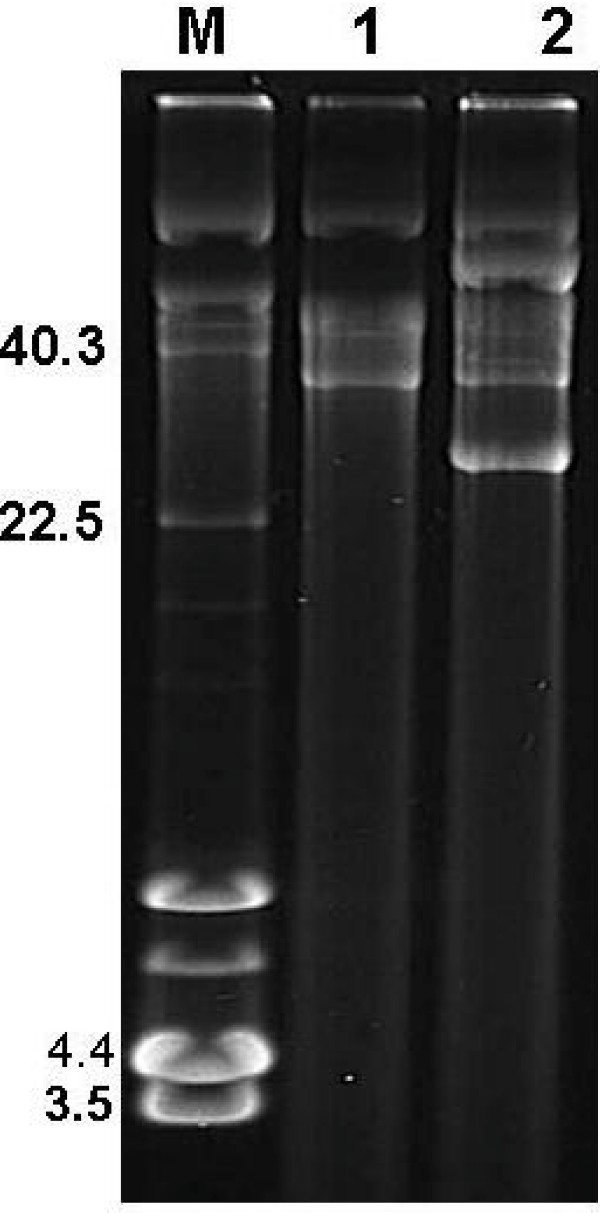
**Plasmid profile of transconjugants derived from the methicillin/mupirocin resistant *S. aureus *(strain A15) from South Africa**. M – WBG 4483 (only the closed circular forms of the plasmids are labelled); Lane 1 – TransMup (resistant to mupirocin); Lane 2 – TransCad (resistant to mupirocin and cadmium).

**Figure 6:**
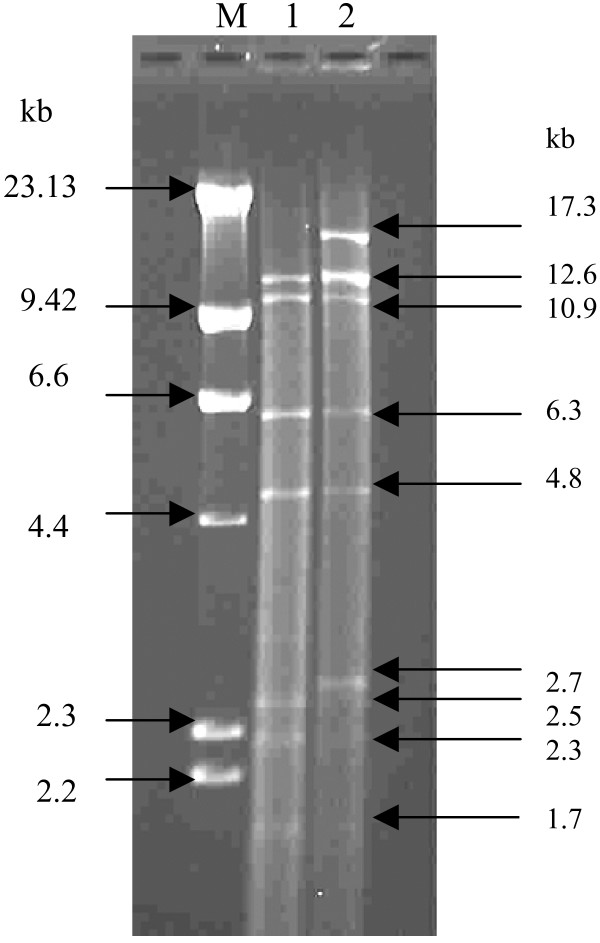
***EcoR*I restriction pattern (plasmids) of transconjugants derived from the methicillin/mupirocin resistant *S. aureus *(A15) strain from South Africa**. M: Molecular weight standard, phage lambda DNA digested with *Hind*III; Lane 1 – TransMup (resistant to mupirocin); Lane 2 – TransCad (resistant to mupirocin and cadmium).

Transconjugants were not observed on mupirocin selection plates when 35 IBA was used as the donor strain. However, transconjugants were obtained on mupirocin selection plates with A15 as the donor strain. Replica plating of 98 transconjugants on BHIA selection plates with cadmium (5 mg/L), erythromycin (5 mg/L) and mupirocin (10 mg/L) yielded one, three and 98 colonies respectively. The susceptibility pattern of the parent and cured strains along with the transconjugants are presented in Table 2. The transfer frequency was calculated as 1.2 × 10^-5 ^and 2 × 10^-8 ^for mupirocin and erythromycin resistance determinants respectively. Transconjugants that were resistant to mupirocin contained a single plasmid of *c*. 38 kb while those resistant to mupirocin and cadmium carried two plasmids of *c*. 38 kb and 25 kb in size (Figure 5). *Eco*RI restriction analysis showed that the plasmid in the mupirocin-resistant transconjugant (TransMup) yielded seven fragments (12.6, 10.9, 6.3, 4.8, 2.5, 2.3 and 1.7 kb) indicating that the size of the mupirocin plasmid was 41.1 kb (Figure 6). The two plasmids in mupirocin and cadmium-resistant transconjugant (TransCad) produced nine *Eco*RI fragments (17.3, 12.6, 10.9, 6.3, 4.8, 2.7, 2.5, 2.3, 1.7 kb) with seven fragments identical to those of the 41.1 kb plasmid in TransMup. The additional 17.3 and 2.7 kb (20 kb) fragments were associated with the second plasmid carrying cadmium resistance in TransCad (Figure 6).

**Table 2:** Susceptibility profile of parent, cured, recipient strains and transconjugants derived from strain A15

Strain	Resistance pattern	MUP MIC (mg/L)	mupA gene
35 IBA (parent)	TET, MU (H)	>1024	+

35 IBA (CUR) – cured strain	TET	0.064	-

A15 (parent)	OX, ERY, CD^Ri^, RF, TET, MU (H)	>1024	+

A15^c ^A (cured strain of A15)	OX, RF, TET, MU (H)	>1024	+

A15^c ^D (cured strain of A15)	OX, ERY, CD^Ri^, RF	0.0125	-

WBG 541 (recipient strain)	RF, FC	ND	-

TransEry (transconjugant of A15)	RF, FC, ERY, CD^Ri^	ND	-

TransMup (transconjugant of A15)	RF, FC, MU (H)	>1024	+

TransCad (transconjugant of A15)	RF, FC, MU (H), Cad	>1024	+

TET – Tetracycline; MU (H) – High-level mupirocin resistance; OX – Oxacillin;

ERY – Erythromycin; CD^Ri ^– Clindamycin (inducible resistance); RF – Rifampicin;

FC – Fusidic acid; Cad – Cadmium; ND – Not determined

## Discussion

This investigation studied mupirocin-resistant *S. aureus *that were isolated in Nigeria and South Africa, using phenotypic and molecular methods. Clinical isolates of mupirocin-resistant *S. aureus *was first reported in 1987 [34], and resistance has frequently been attributed to the clinical use of mupirocin over extended periods [23] or in areas of highly concentrated application, such as dermatology or burns units [35,36]. Mupirocin is prescribed and administered for the treatment of MRSA infections and *S. aureus *nasal colonization among hospital patients in KZN, and it appears that the extensive use of the agent in health care institutions has contributed to the emerging trend of *S. aureus *resistance. Typing based on phenotypic and genotypic methods play an important role in understanding the epidemiology of MRSA and evaluating the effectiveness of infection control and antimicrobial prescribing measures [37]. In this study, two main PFGE types (A and B) were identified among the low-level mupirocin resistant strains obtained from six health care institutions in the KZN province of South Africa. The strains in pulsotype A were identified in two health care institutions located in Durban and a health care facility in Pietermaritzburg, Greytown and Empangeni, while pulsotype B was noted in two health care institutions in Durban and in Eshowe. Some degree of correlation was observed with the three typing methods. The low-level mupirocin-resistant strains with a PCR-RFLP pattern of 81, 567 bp belonged to the clone B by PFGE typing. Moreover, MRSA strains with PCR-RFLP pattern of 81, 324, 405 bp and 324, 405 bp had similar antibiotic resistance pattern and belonged to the dominant clone A. Hence, this study has demonstrated clonal dissemination of multiresistant MRSA strains exhibiting low-level resistance to mupirocin in health institutions in KZN province of South Africa. The emergence and spread of low-level mupirocin-resistant *S. aureus *should be of concern among health care workers in South Africa because of recent reports on the increase in treatment failure rates for nasal decolonization of MRSA due to the emergence of low-level mupirocin resistance [16-18].

Plasmid-mediated resistance to antimicrobial agents among pathogenic bacteria constitutes a major clinical and economic problem worldwide. In this study, the genetic location of the high-level mupirocin resistance determinant was resolved in two isolates by plasmid analysis, involving curing and conjugation experiments. Three features were identified in the transfer experiments and plasmid analysis. The first feature was the transfer of the 41.1 kb plasmid encoding high-level mupirocin resistance. High-level mupirocin resistance has been found in self-transmissible and non-self transmissible plasmids in different countries [4,38-40], and this study has demonstrated what appears to be the first report of a conjugative mupirocin resistance plasmid in South Africa. The conjugative transfer of plasmids mediating high-level mupirocin resistance may involve the co-transfer of small non-conjugative plasmids encoding resistance to tetracycline and chloramphenicol [22,23,40,41] and large plasmids encoding resistance to penicillin [42]. The transfer of resistance determinants mediating mupirocin and triclosan resistance in MRSA has also been reported [43]. The second feature was the conjugative transfer of a 41.1 kb plasmid mediating mupirocin resistance along with the co-transfer of a plasmid encoding resistance to cadmium (Table 2; Figure 5). This observation suggests that the 41.1 kb plasmid belong to a class of conjugative plasmids that could be co-transferred with other resistance determinants. The third feature was the transfer of a 2.3 kb plasmid mediating erythromycin resistance. Although our observations on plasmid analysis and conjugation experiments are preliminary due to the number of isolates studied, the demonstration of conjugative transfer of the mupirocin resistance plasmid and its co-transfer with other resistance plasmids clearly supports the judicious use of this topical antibiotic in health institutions in South Africa. Hence, urgent measures need to be taken to prevent clonal dissemination of the mupirocin/methicillin resistant *S. aureus *in KZN, South Africa. Future work include investigating the nature of the plasmid encoding mupirocin resistance in 35 IBA and P1929, the full sequence and comparison of the *mupA *gene with other sequences in the GenBank. This could probably provide new insights on the evolution of the *mupA *gene in *S. aureus*.

## Conclusion

This study has demonstrated the clonal dissemination of multi-resistant MRSA strains exhibiting low-level resistance to mupirocin in KZN and the emergence of high-level mupirocin resistant *S. aureus *in Nigeria and South Africa. Although the high-level mupirocin resistant *S. aureus *strains from the two countries are genetically unrelated, the ability of the 41.1 kb mupirocin plasmid to transfer the resistance determinant indicate the potential for spread to mupirocin-susceptible MSSA and MRSA isolates under the selective pressure of mupirocin. It is also recommended that routine testing of MSSA and MRSA for mupirocin resistance be conducted even in facilities where mupirocin is not administered. This will facilitate the early detection of resistance and assist in the control and spread of mupirocin-resistant *S. aureus*.

## Competing interests

The authors declare that they have no competing interests.

## Authors' contributions

AOS participated in the collection, susceptibility pattern and characterization of the mupirocin-resistant isolates. EEU planned the plasmid analysis and JL supervised the project. All authors participated in the preparation of the final manuscript.

## Pre-publication history

The pre-publication history for this paper can be accessed here:

http://www.biomedcentral.com/1471-2334/9/10/prepub
